# Effect of 3D Slicer Preoperative Planning and Intraoperative Guidance with Mobile Phone Virtual Reality Technology on Brain Glioma Surgery

**DOI:** 10.1155/2022/9627663

**Published:** 2022-05-24

**Authors:** Jun Liu, Xiaodong Li, Xueping Leng, Bo Zhong, Yanhong Liu, Liang Liu

**Affiliations:** ^1^Department of Neurosurgery, The Second Affiliated Hospital of Wannan Medical College, Wuhu 241000, Anhui, China; ^2^Department of Neurosurgery, Pingyi County Hospital of Traditional Chinese Medicine, Linyi 273300, Shandong, China; ^3^Department of Neurosurgery, The Affiliated Hospital of Qingdao University, Qingdao 266000, Shandong, China; ^4^Department of Neurosurgery, Yidu Central Hospital, Weifang 262500, Shandong, China; ^5^Department of Pain Treatment, Yidu Central Hospital, Weifang 262500, Shandong, China; ^6^Department of Radiology, Nanjing Pukou District Central Hospital, Nanjing 211899, Jiangsu, China

## Abstract

**Objective:**

To explore the effect of 3D Slicer preoperative planning and intraoperative guidance with mobile phone virtual reality (VR) technology on brain glioma surgery.

**Methods:**

By means of retrospective study, the data of 77 brain glioma patients treated in the neurosurgery departments at The Second Affiliated Hospital of Wannan Medical College and Xinhua Hospital Affiliated to Shanghai Jiao Tong University School of Medicine from January 2015 to January 2022 were analyzed, and the patients were divided into the experimental group (EG, *n* = 38) and the control group (CG, *n* = 39) according to the surgical modalities. Before surgery, all patients received positron emission tomography-computed tomography (PET/CT) scanning and magnetic resonance imaging (MRI) examination. For patients in EG, the DICOM format images acquired from PET-CT and MRI examinations were imported with the 3D Slicer software for 3D visual fusion reconstruction, acquiring VR images, and developing detailed preoperative planning. Then, the reconstructed images were imported into the Sina software on a mobile phone, and the surgery was performed with the assistance of VR technology; for patients in CG, traditional 2D images were used for tumor contour drawing by the subjective visual method, and the craniotomy was performed under a traditional microscope. Patients' surgery indicators and Karnofsky Performance Scale (KPS) scores were compared between the two groups.

**Results:**

The number of cases with total resection, rate of total resection, hospital stay after surgery, and surgery time were significantly better in EG than in CG (*P* < 0.05); after treatment, the KPS score was significantly higher in EG than in CG (75.66 ± 4.01 vs 65.36 ± 5.23, *P* < 0.001).

**Conclusion:**

Combining 3D Slicer preoperative planning with intraoperative mobile phone VR technology can promote the accuracy of brain glioma surgery, which is conducive to effectively removing tumors while protecting patients' neural function.

## 1. Introduction

Brain glioma, the most common malignant primary brain tumor in clinic, is induced by a combination of genetic high-risk factors and environmental factors, and its incidence accounts for approximately 40.49% of all brain tumors and adult incidence is 8 in 100,000 [1]. Since the disease occurs in the brain, patients' symptoms and signs are closely related to the function of the affected brain regions. In addition to headaches, nausea, and blurring of vision caused by space-occupying effects, optic gliomas trigger visual loss, spinal gliomas trigger pain in the extremities, and central area gliomas cause sensory disturbances [2, 3], so the location of the lesion can be initially inferred based on the presenting symptoms and signs, but the final qualitative diagnosis still relies on other tests such as magnetic resonance (MR) examination and pathological examination. MR is one of the most common diagnostic methods for brain glioma, and the optimization and upgrade of 3D Slicer in recent years has gradually broadened its application field. Therefore, magnetic resonance imaging (MRI) has become the most dominant imaging modality in preoperative planning and evaluation of brain tumor surgery at the current stage [4, 5]. 3D Slicer is an image analysis and processing platform jointly developed by Harvard University and Massachusetts Institute of Technology, which can combine the images acquired from imaging examinations, such as MRI and CT, in the way of image fusion to build multimodal virtual 3D visualization models [6, 7], thus exhibiting more clear tumor contours and surrounding tissues, facilitating physicians to dynamically observe intracranial lesions and intuitively learn the relationship between tumor tissue and surrounding structures, and providing a scientific basis for the development and execution of surgical protocols.

Studies at home and abroad have shown that planning neurosurgical protocol with the aid of 3D Slicer before surgery is beneficial for elevating the tumor resection rate and protecting the neurological function of patients, and is of great significance for improving patient outcomes. With the breakthrough progress of virtual reality (VR) technology, the intraoperative guidance effect of 3D Slicer for neurosurgery has also been gradually demonstrated. By importing the 3D visualization reconstructed image obtained by this software into mobile phone VR software, physicians are able to perform image-head fusion during brain surgery while wearing VR glasses [8, 9], and capture the tumor information that is difficult to recognize with the naked eye, thereby greatly improving medical image registration and increase the precision of body surface localization [10], and overcoming the disadvantages of completely relying on surgeons' experience in traditional surgery. At present, several studies have explored the application value of 3D Slicer in preoperative planning, but there is still a gap in research on the application of 3D Slicer in glioma surgery. Based on this, the effect of preoperative planning with 3D Slicer and intraoperative guiding brain glioma surgery with mobile VR technology was explored herein.

## 2. Materials and Methods

### 2.1. Study Design

This retrospective study was conducted in the neurosurgery departments at The Second Affiliated Hospital of Wannan Medical College and Xinhua Hospital Affiliated to Shanghai Jiao Tong University School of Medicine from January 2015 to January 2022, aiming to explore the effect of 3D Slicer preoperative planning and intraoperative guidance with mobile VR technology on brain glioma surgery.

### 2.2. Inclusion and Exclusion Criteria

Inclusion criteria are as follows: (1) The patients were diagnosed with brain glioma after pathological examination and imaging examination [11]; (2) the patients met the surgical indications; (3) the patients were treated in our hospital for the whole course; (4) the patients did not have obvious abnormalities in liver function and routine blood indexes; (5) the patients were at least 18 years old; (6) the estimated survival of the patients was over 6 months.

Exclusion criteria are as follows: (1) The patients were complicated with severe cardiovascular and cerebrovascular diseases; (2) the estimated survival of the patients was less than 6 months; (3) the patients had surgical contraindication; (4) the patients had poor body tolerance; (5) the patients were complicated with coagulation dysfunction or other malignant tumors; (6) the patients were unable to communicate with others due to severe mental disorder, speech disorder, and disturbance of consciousness; (7) the patients were allergic to the drugs involved in the study; (8) the patients had contraindications of CT and MRI examinations; (9) the patients were pregnant or lactating women.

### 2.3. Grouping

The data of brain glioma patients treated in the neurosurgery departments at The Second Affiliated Hospital of Wannan Medical College and Xinhua Hospital Affiliated to Shanghai Jiao Tong University School of Medicine from January 2015 to January 2022 were retrospectively analyzed, and 77 patients were selected as the subjects. According to the surgical modalities, the patients were divided into the experimental group (EG, *n* = 38) and control group (CG, *n* = 39). In EG, there were 25 males and 13 females, the patient's mean age was (55.98 ± 13.70) years, and according to the WHO classification of brain gliomas, EG included 1 grade I case, 6 grade II cases, 10 grade III cases, and 21 grade IV cases; and in CG, there were 23 males and 16 females, the patient's mean age was (57.15 ± 12.97) years, and according to the WHO classification of brain gliomas, CG included 1 grade I case, 13 grade II cases, 6 grade III cases, and 19 grade IV cases. No statistical between-group differences in patients' baseline data were observed (*P* > 0.05), presenting the study value.

### 2.4. Moral Consideration

The study met the principles in the *World Medical Association Declaration of Helsinki (2013)* [12], and the patients understood the study objective, meaning, contents and confidentiality and signed the informed consent.

### 2.5. Methods

#### 2.5.1. Imaging Examination

Before surgery, all patients received a routine head MRI (Siemens Healthcare GmbH; NMPA Registration (I) no. 20193060345) examination. The patients were in the supine position with both hands placed on either side of the body, the standard 8-channel head coil was selected for routine T_I_WI and T_2_WI scanning, respectively, and then, the contrast agent gadopentetate dimeglumine (manufacturer: Beijing Beilu Pharmaceutical Co. Ltd.; NMPA approval no. H10860002) was administered via intravenous injection at a rate of 2 mL/s for enhanced T_I_WI scanning. Most patients accepted magnetic resonance angiography (MRA) at the same time.

Due to the limited hardware condition of the hospital, the PET-CT examination was conducted in other hospitals. Within 24 hours before examination, all types of drugs affecting the central nervous system (CNS) were contraindicated. During examination, the patients were in the supine position and lied quietly for 30 min, and tried not to be disturbed by things or sounds. The 11 C-methionine (^11^C-MET) was used as the tracer with the injection dose of 0.15–0.20 mCi/kg for the PET-CT (Siemens Medical Solutions USA; NMPA Registration (I) no. 20173062264) scanning, which was performed in strict accordance with the baseline, slice thickness and gap of MRI axial scan. The images underwent regular attenuation correction, reconstruction and quantitative analysis.

#### 2.5.2. Development of Surgical Plan

For patients in CG, the tumor contour was drawn on the cross-sectional images of different sequences by the subjective visual method, and obvious lesion enhancement in the enhanced T_I_WI sequence was identified as the lesion area; if the enhanced T_I_WI sequence had no enhancement or showed punctate enhancement, the high signal area of the T_2_WI sequence was the lesion area. As for the PET-CT images, the region significantly higher than the surrounding normal gray matter was regarded as the tumor focus and used to outline the tumor contour. According to the outlined image, the corresponding surgical plan was developed.

For patients in EG, the MRI and PET-CT data in the DICOM format were imported into 3D Slicer to complete the 3D reconstruction of the skull, bone of cerebral cranium, blood vessels, and glioma through the Editor program, and then, image fusion was performed by the registration program. By adjusting the transparency, the tumor tissue, blood vessels and nerves could appear more clearly for the physicians to make preoperative planning and design the surgery plan according to the reconstructed images. For the cases undergoing surgery guided by mobile phone VR intraoperatively, the images obtained from preoperative reconstruction were imported into the Sina software on the mobile phone. During surgery, the accuracy of the reconstructed images was tested again with body surface landmarks of the head such as nasal tip and inner canthus.

#### 2.5.3. Mobile Phone VR Technology-Guided Brain Glioma Surgery

Patients in the two groups underwent surgery with the help of microscope under general anesthesia. According to the lesions shown in the image data, including the size of skin incision and bone flap, the best surgical path was selected, and the adjacent important anatomical structures were avoided. Under the guidance of Sina, blocks were established for patients in EG through the surgical information, the images of the same and similar levels were automatically matched, and at the same time the tumor boundary was outlined and the surgery area was located. During the surgery, the residual tumor was analyzed according to the anatomical markers around the surgery area. If residual tumor was found, the physicians performed the next resection according to the preoperative planning.

### 2.6. Observation Standards

1. Surgical indicators. Including the number of cases with total resection, rate of total resection, hospital stay after surgery, and surgery time.
2. Karnofsky performance scale (KPS). The KPS scale [13] was used to evaluate patients' quality of life (QOL) two months after surgery. The scale was divided into 10 scoring grade, which ranged from normal functioning without symptoms and signs (100 points) to dead (0 point), with higher scores indicating better QOL.

### 2.7. Statistical Processing

In this study, the data processing software was SPSS20.0, the two kinds of picture drawing software were GraphPad Prism 7 (GraphPad Software, San Diego, USA) and 3D SLICER 4.10.2, the items included were enumeration data and measurement data, the methods used were the *X*^2^ test and *t*-test, and differences were considered statistically significant at *P* < 0.05.

## 3. Results

### 3.1. Comparison of Patients' Surgical Indicators

**Figure 1:**
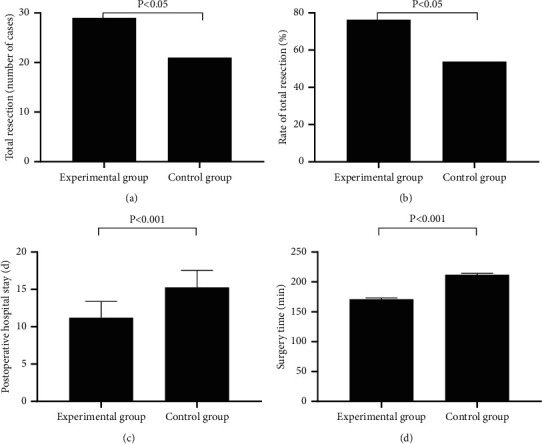
Comparison of patients' surgical indicators. (a) The number of cases with total resection; (b) The rate of total resection; (c) The postoperative hospital stay; (d) The surgery time.

Figure 1 showed that the surgical indicators were significantly better in EG than in CG (*P* < 0.05).

In EG, the number of cases with total resection was 29, the rate of total resection was 76.3%, the postoperative hospital stay was (11.21 ± 2.18) d, and the surgery time was (171.21 ± 2.08) min; and in CG, the number of cases with total resection was 21, the rate of total resection was 53.8%, the postoperative hospital stay was (15.28 ± 2.28) d, and the surgery time was (212.26 ± 2.27) min.

### 3.2. Comparison of Patients' KPS Scores

**Figure 2:**
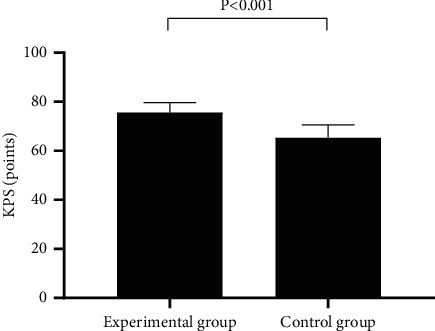
Comparison of patients' KPS scores (*x* ± *s*). In Figure 2, the horizontal axis indicated EG and CG, and the vertical axis indicated the KPS score (points).

Figure 2 showed that after treatment, the KPS score was significantly higher in EG than in CG (75.66 ± 4.01 vs 65.36 ± 5.23, *P* < 0.001).

## 4. Discussion

Brain gliomas are tumors in CNS originating from glial cells, which are classified into low-grade (I and II) gliomas and high-grade (III and IV) gliomas by the WHO based on pathological manifestations [14]. The survival of patients with early to mid-term brain glioma can be prolonged through surgery, but the surgical effect is affected by the extent of tumor resection. In the past, traditional craniotomy has the defect of low macroscopic resolution, making it difficult to distinguish the boundary between glioma and normal brain tissue intraoperatively, so the total resection rate is low and the normal brain tissues around the lesion are susceptible to damage [15, 16]. Maximal resection of gliomas while protecting normal brain tissues is key to improving patient outcomes, so it is extremely critical to clarify the pathological extent of gliomas. Because gliomas show infiltrative growth and no obvious boundary, and their occurrence site is relatively specific, it is difficult to realize the biological total resection in practice. Generally, total resection is clinically divided into macroscopic total resection, microscopic total resection, and imaging total resection, among which the imaging total resection is the closest to the effect of biological total resection [17, 18], and therefore, the improvement of total resection ability in imaging means that the total resection effect is optimized.

With the optimization and upgrade of medical imaging technology, image fusion technology has been applied in the assessment before and after brain gliomas surgery. This multimodal imaging modality uses computer technology to digitally and comprehensively process the image information acquired from various imaging examinations for multivariate data synergistic use and then generates a brand-new imaging after spatial registration [19, 20]. It is beneficial for physicians to understand the comprehensive information of lesion tissues to develop more rational treatment regimens or evaluate the therapeutic effects. 3D Slicer is a computer software based on image fusion technology, which can reconstruct the preoperatively acquired imaging data from MRI, CT, PET-CT, etc. to provide massive and precise stereo anatomical information [21], and enable the physicians to visually observe the spatial positional relationship between tumor and surrounding tissues on 3D images by adjusting transparency, thereby avoiding important anatomical locations, blood vessels, nerves, etc. for preoperative planning [22, 23]. Not only that, with preoperative 3D reconstruction and surgical simulation, physicians can be more clear about the risks that may arise during surgery and explain the surgical protocol to patients more easily while developing a corresponding emergency plan. During the actual procedure, physicians can import the images acquired by 3D Slicer into the mobile phone, overlap the virtual 3D intracranial imaging with the realistic head with the help of mobile VR [24] so that the anatomical position of the patients' head is fused with the marked position, thus improving the precision of the procedure and reducing the damage to the patients' normal nerve. With the assistance of 3D Slicer, physicians can perform the procedure more smoothly, and therefore the surgical indicators of EG were significantly better than those of CG (*P* < 0.05). Besides that, 3D Slicer is also able to effectively overcome the drawbacks of failing to identify white matter fiber tract structure by conventional imaging, it can exhibit the arrangement of cerebral white matter and enable the visualization of fiber tracts with the help of diffusion tensor imaging and fiber tractography, which is beneficial for avoiding intraoperative secondary damage, protecting the neurological function of patients, and accelerating the postoperative recovery process of patients and guaranteeing their QOL, so patients in EG had a better QOL after surgery. Yahanda et al. applied image fusion technology in the evacuation of intracranial hematoma and found that multimodal fusion imaging with 3D reconstruction has significant guidance for surgical planning, indicating that 3D reconstruction could effectively improve the success rate of clinical surgery [25]. However, it should be noted that 3D Slicer cannot simulate the real tumor manipulation. Although this study concluded that the patients in EG had better surgical indicators, this software still cannot be systematically applied in brain glioma surgery, and more samples are needed to confirm the application effect of 3D Slicer and provide more scientific evidence for clinical usage.

Glioma occupies an important role in brain malignancy, which has a significant negative impact on the normal life of patients. Early surgery can improve patient outcomes and their quality of life. The study found that combining 3D Slicer preoperative planning with intraoperative mobile phone VR technology can promote the accuracy of brain glioma surgery, which is conducive to effectively removing tumors while protecting patients' neural function and reducing the medical burden on brain glioma patients.

## Data Availability

The data used to support the findings of this study are available on reasonable request from the corresponding author.
